# Novel Noninvasive Brain Disease Detection System Using a Facial Image Sensor

**DOI:** 10.3390/s17122843

**Published:** 2017-12-08

**Authors:** Ting Shu, Bob Zhang, Yuan Yan Tang

**Affiliations:** Department of Computer and Information Science, Avenida da Universidade, University of Macau, Taipa, Macau 999078, China; yb57406@umac.mo (T.S.); yytang@umac.mo (Y.Y.T.)

**Keywords:** image sensor, brain disease, noninvasive detection system, facial key block analysis, ProCRC, medical biometrics

## Abstract

Brain disease including any conditions or disabilities that affect the brain is fast becoming a leading cause of death. The traditional diagnostic methods of brain disease are time-consuming, inconvenient and non-patient friendly. As more and more individuals undergo examinations to determine if they suffer from any form of brain disease, developing noninvasive, efficient, and patient friendly detection systems will be beneficial. Therefore, in this paper, we propose a novel noninvasive brain disease detection system based on the analysis of facial colors. The system consists of four components. A facial image is first captured through a specialized sensor, where four facial key blocks are next located automatically from the various facial regions. Color features are extracted from each block to form a feature vector for classification via the Probabilistic Collaborative based Classifier. To thoroughly test the system and its performance, seven facial key block combinations were experimented. The best result was achieved using the second facial key block, where it showed that the Probabilistic Collaborative based Classifier is the most suitable. The overall performance of the proposed system achieves an accuracy −95%, a sensitivity −94.33%, a specificity −95.67%, and an average processing time (for one sample) of <1 min at brain disease detection.

## 1. Introduction

The brain as the control center of the body governs our thoughts, memory, speech, and movement [1]. Brain Disease (BD) includes any conditions or disabilities that affect the brain [2]. When the brain is damaged, it affects our memory, sensation, and even our personality [3]. The damages caused by this disease can severely affect a person’s daily life [2]. The World Health Organization (WHO) [4] estimated that BD attributed deaths in relation to the total deaths worldwide stood at 11.67% and 11.84% in 2005 and 2015, respectively, and is set to increase to 12.22% in 2030.

There are many categories of BD, such as: infections, stroke, trauma, seizures, tumors, and so on [5]. For the various categories, doctors have many different ways to diagnosis BD. There are three main traditional BD diagnostic methods: Computed Tomography (CT) [6], Magnetic Resonance Imaging (MRI) [7], and Functional MRI (fMRI) [8]. A CT scan of the head is taken using a special X-ray equipment to produce multiple images of the brain [6]. However, this diagnostic method has some drawbacks. First, it requires the examinee to remain still while the image is taken for an amount of time ranging from 30 s to 10 min (depending on the examination). Secondly, the CT scan subjects the individual being examined to small doses of radiation. Furthermore, the procedure can be considered invasive if blood vessels in the brain are to be imaged, since it requires the injection of a solution to highlight these areas. As for MRI scanners, radio waves, a powerful magnetic field, and field gradients are applied to generate images of the brain [7]. fMRI on the other hand is similar to MRI, where it uses the results of the MRI to measure small variations that are of the metabolic nature taking place in the brain’s active region [8]. When compared with a CT scan, both MRI and fMRI scans normally take longer to complete. In addition, due to use of field gradients, the sound produced by an MRI/fMRI scan can be very loud.

According to the information given above, BD is a serious illness that affects the world’s population. However, its current diagnostic methods are either invasive or non-patient friendly or both. Therefore, it is necessary to develop an accurate, convenient, effective, non-invasive, and patient friendly BD diagnostic system.

In this paper, we mainly focus on brain injuries, especially Cerebral Infarction (also named stroke). In our brain disease dataset, 67.23% is composed of stroke patients, while the other 32.77% consists of other brain related illnesses combined into a single class (refer to Section 3). A Cerebral Infarction is an area of necrotic tissue in the brain resulting from a blockage or narrowing in the arteries supplying blood and oxygen to the brain [9]. Both the blockage and narrowing of the arteries can influence the normal supply of the blood and oxygen to the brain [10]. The abnormal supply of the blood and oxygen to the brain can result in the patient having different facial color and texture complexion than someone that is healthy. Therefore, some researchers [11,12,13,14,15,16] have carried out many related works to diagnose disease based on facial color and texture features.

In 2008, Kim et al. proposed a method to conduct the color compensation of a facial image based on the analysis of facial color to assist the doctor in diagnosing heart disease [11]. Using a computerized method to detect diabetes mellitus was proposed in [12,13,14,15]. In 2015, Shu et al. developed a system to detect the health status of an individual through a noninvasive computerized method [16]. Ťupa et al. [17] proposed a method to recognize Parkinson’s disease through motion tracking and gait feature estimation. These proposed methods [11,12,13,14,15,16,17] and their experimental results proved that a convenient and user friendly system taking advantage of information science can be developed to detect an individual’s health status and even a specific disease effectively and efficiently. On the other hand, Procházka et al. [18,19] proved that an efficient sensor can help detect gait disorders and analyze breathing and heart rate, respectively.

Sparse Representation (SR) has been used in many applications and obtained great performances [20,21,22,23]. These applications include image classification [20], image denoising [21], image alignment [22], and image super-resolution [23]. In 2011, Zhang et al. discussed whether SR is necessary in face recognition, as it uses $l_{1}$-norm, which is time-consuming [24]. According to their experimental results, they proved that SR is not necessary and proposed the Collaborative Representation based Classifier (CRC) [24] applied in face recognition, achieving a noticeable performance and was much faster than the SR based Classifier (SRC) [20]. In 2016, Cai et al. combined probabilistic theory and CRC together to develop a probabilistic CRC (ProCRC) used in pattern recognition [25].

These computerized noninvasive disease detection methods [11,12,13,14,15,16] and ProCRC inspired us to develop a convenient and patient friendly noninvasive BD detection system (NBDS). The system employs a noninvasive facial image sensor to take an image of an individual. From four regions of the facial image, four key blocks are next automatically extracted from these regions. For each facial key block, a color gamut consisting of six main color centroids are used to extract the color features. Using the facial key block color features, ProCRC is modified and applied to detect BD and healthy samples. NBDS is trained and tested on a new dataset including 119 BD patients and 595 Healthy (H) individuals.

## 2. Noninvasive BD Detection System (NBDS)

This section describes the NBDS based on facial key block color feature analysis. Firstly, the system structure is given followed by a description of the facial image sensor. Next, automatic facial key block extraction is discussed. Then, the color features from each facial key block is given. Lastly, we introduce how ProCRC is applied to NBDS.

### 2.1. NBDS Structure

**Figure 1:**
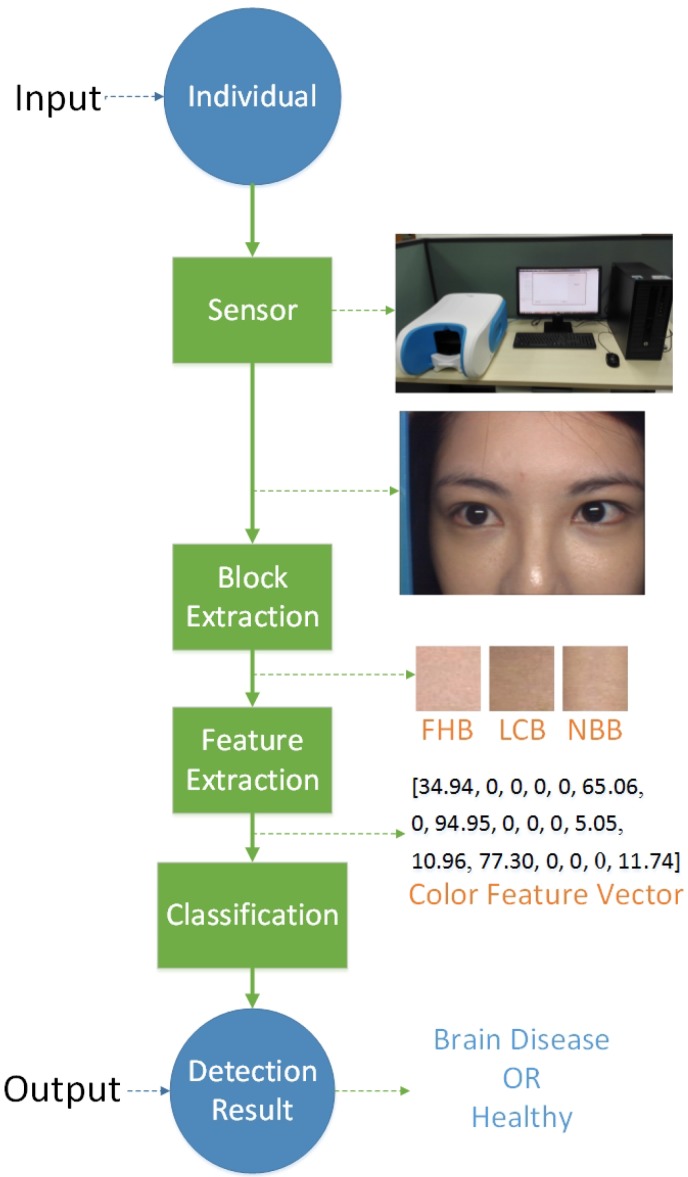
Noninvasive Brain Disease Detection System (NBDS) structure figure. NBDS consists of four main components: (**a**) Sensor; (**b**) Block Extraction; (**c**) Feature Extraction; and (**d**) Classification.

The NBDS structure is illustrated in Figure 1, where the circles signify the input and output of this system and the rectangles represent the components of NBDS. On the right side of the system diagram, one corresponding example of each component is given. In Figure 1, the first rectangle named *Sensor* is the facial image device. More details about this sensor can be found in Section 2.2. Using this sensor, the data captured is a facial image of a person. For the second component (*Block Extraction*), the data (facial image) is converted into four facial key blocks extracted automatically. How to extract the blocks is given in Section 2.3. The third rectangle indicates *Feature Extraction* and the data processed by this procedure is morphed to a feature vector. This feature vector consists of three facial block feature vectors, as each key block has six feature values, and they are concentrated together into one vector with 18 dimensions. Section 2.4 describes the facial key block color feature extraction. Using this feature vector, a *Classifier* is used to decide which class the sample belongs to. In the proposed system, the ProCRC is used as the classifier and more information about it is presented in Section 2.5.

### 2.2. Facial Image Sensor

**Figure 2:**
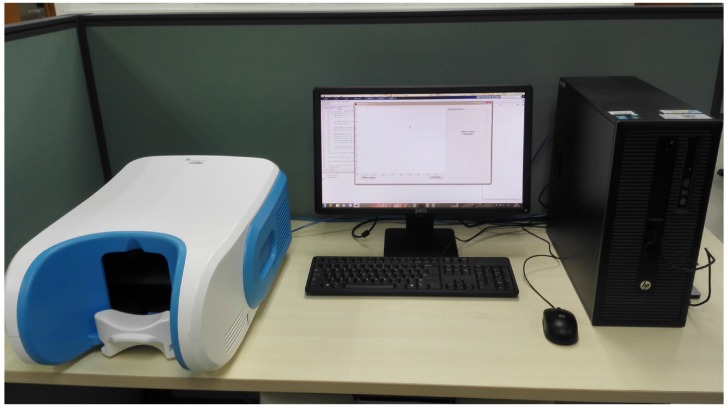
Facial image capture device.

**Figure 3:**
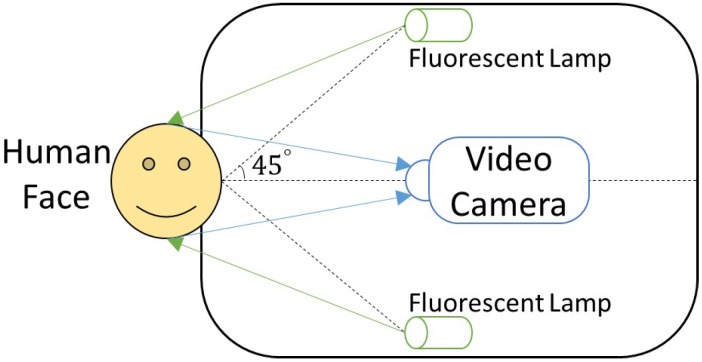
Facial image capture device light path.

In order to achieve automatic noninvasive brain disease detection through facial image analysis, its capture and representation is vital. To ensure unbiased feature extraction and analysis in the subsequent steps, facial images from those suffering from brain disease must be captured and depicted in an accurate way under a standardized setting. A uniquely designed facial image capture device with calibration was developed to resolve this issue (see Figure 2) in collaboration with the Harbin Institute of Technology Shenzhen Graduate School. The dimensions of the capture device are 390 mm (width) × 220 mm (height) × 600 mm (depth). The primary module of this device is a SONY ( Tokyo, Japan) three separate charge-coupled devices (3-CCD) video camera (DXC-390P), which is a high-end industrial camera able to acquire 25 images per second. The camera is compact and lightweight, reducing the overall weight of the device and making it portable. Furthermore, the video camera incorporates Sony’s new 10-bit digital signal processing (DSP) technology that enables a variety of enhancement features and increases picture reliability. In addition to this, the camera also produces high quality pictures that are necessary to accurately represent each captured facial image. Other less expensive cameras were experimented, but the results were not suitable. The schematic diagram of the viewing angle and imaging path of the device is shown in Figure 3. By placing the camera in the center of the device, two fluorescent lamps are situated on both sides. The angle between the incident light and emergent light is $45^{\circ }$, as recommended by the Commission Internationale de l’Eclairage (CIE) [26].

**Figure 4:**
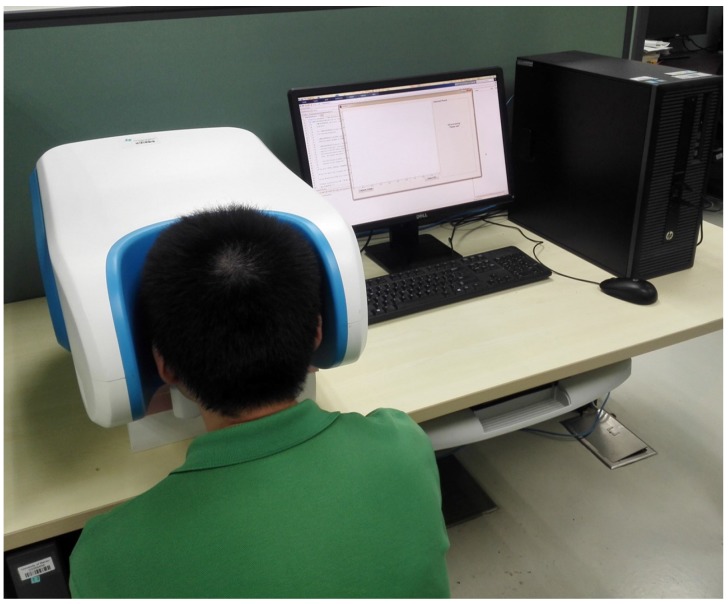
A simulation of an individual using this device.

During data collection, an individual will sit in front of the device resting his/her chin on the chin rest while looking straight into the camera. This allows us to fix the position of their face. With the device connected to a workstation, an operator will control the device’s functions. A simulation of an individual using this device is illustrated in Figure 4.

**Figure 5:**
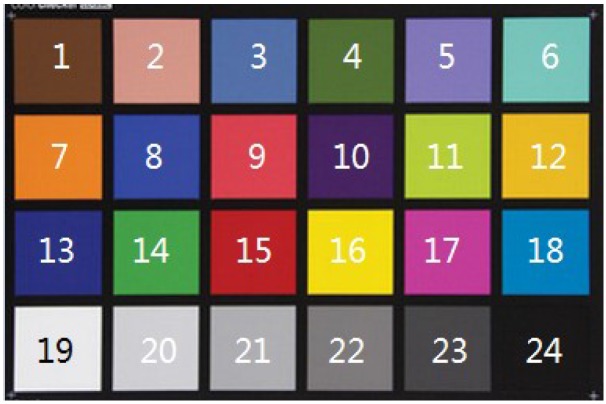
Munsell color checker.

In order to portray the color images in a precise way so as to facilitate quantitative analysis, a color correction procedure [27] is performed before feature extraction and classification. This will eliminate any variances in the color images caused by variations of illumination and device dependency. Thus, it allows images taken in different environments to be equally compared to each other. An algorithm developed by [27] was applied and adapted from its original use in tongue images to correct the facial images. Using the Munsell Color Checker (refer to Figure 5), a polynomial-based regression method was utilized to train the correction model based on the corresponding values of a reference training set. Hence, in this correction model, uncorrected facial images can be corrected, and rendered to be in standard Red Green Blue (sRGB) color space.

### 2.3. Key Block Extraction

**Figure 6:**
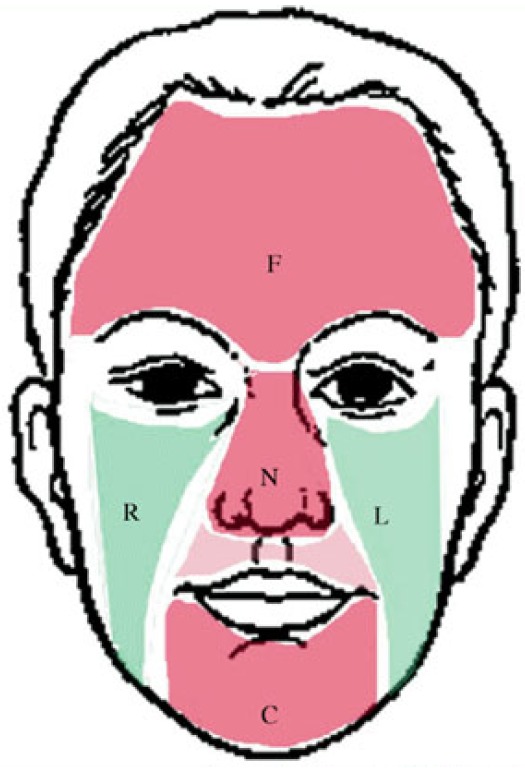
Facial region example.

It is well known that the face can be segmented into five regions based on the positions of the five organs. An example of the five facial regions is illustrated in Figure 6 [28]. The first region is on the forehead below all the facial organs. The left and right cheeks are the second and third regions located below the left and right eyes, respectively. The fourth region covers the nose. The final region comes from the chin. Sometimes, there is facial hair on the chin for men; therefore, the first four regions (five regions except the chin region) are applied in our system.

According to Figure 6, the four region shapes and sizes are different from each other, which makes it difficult for the system to process. Hence, we extract a center key block from each facial region.

**Figure 7:**
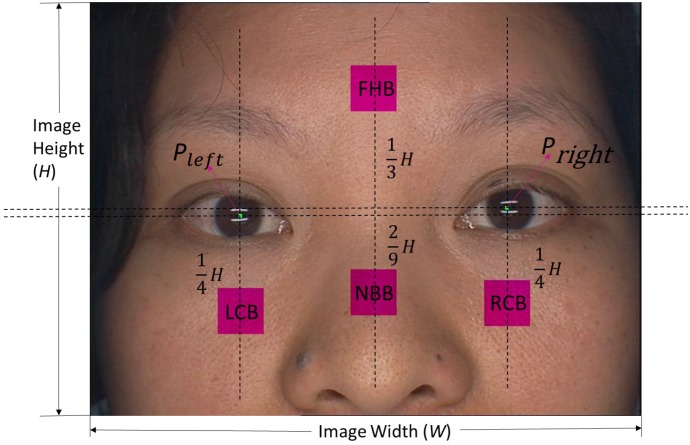
Four facial key block positions. The purplish pink blocks represent the four facial key blocks, where the text in the center of each block is its corresponding name. The two small green patches indicate the two pupil positions.

In order to extract the four facial key blocks automatically, the two pupils are first located using the Canny Edge Detection method [29]. Based on the two pupil positions, four facial regions are segmented. From the center of each region, a key block size of s × s is extracted. Figure 7 shows the four facial key block positions based on the two pupil locations. In order to show the four facial key blocks conveniently, four short names are used to denote them. FHB (forehead block) is the name for the first region key block; the second and third region key blocks are named LCB (left cheek block) and RCB (right cheek block), respectively; the final key block from the fourth facial region uses NBB (nose bridge block) as its short name.

The center positions of the two pupils are denoted as $P_{left}$: $\left(x_{left},\;y_{left}\right)$ (left) and $P_{right}$: $\left(x_{right},\;y_{right}\right)$ (right). Based on $P_{left}$ and $P_{right}$, the four facial key block center positions are calculated through Equations (1)–(4):

(1) $$P_{\mathrm{FHB}}=\left(\frac{x_{left}+x_{right}}{2},\;\frac{y_{left}+y_{right}}{2}+\frac{1}{3}H\right),$$

(2) $$P_{\mathrm{LCB}}=\left(x_{left},\;y_{left}-\frac{1}{4}H\right),$$

(3) $$P_{\mathrm{NBB}}=\left(\frac{x_{left}+x_{right}}{2},\;\frac{y_{left}+y_{right}}{2}-\frac{2}{9}H\right),$$

(4) $$P_{\mathrm{RCB}}=\left(x_{right},\;y_{right}-\frac{1}{4}H\right),$$

where $P_{i^{th}\;\mathrm{key}\;\mathrm{block}\;\mathrm{name}}$ denotes the position of the $i^{th}$ key block, such as $P_{\mathrm{FHB}}$ is the position of FHB; and *W* and *H* are the width and height of the facial image, respectively.

### 2.4. Color Feature Extraction

This subsection introduces the extraction of color features from the four facial key blocks. In order to represent the facial key block colors, a color space is required, where CIEXYZ has been found to be suitable [30,31,32]. Since, the facial key block colors are already in RGB, it is next converted into CIELAB. Finally, the facial key block colors are transformed from CIELAB into the CIEXYZ color space. The CIEXYZ color space was created by the CIE in 1931 [33]. The CIEXYZ was the first color space to define the quantitative links between distributions of wavelengths in the electromagnetic visible spectrum, and the physiological perceived colors in human color vision. In 1948, Hunter Richard Sewall [34] created the CIELAB color space, which was derived from CIEXYZ. The CIELAB color space describes mathematically all perceivable colors in the three dimensions “L” for lightness and “a” and “b” for the color opponents green-red and blue-yellow, respectively.

**Figure 8:**
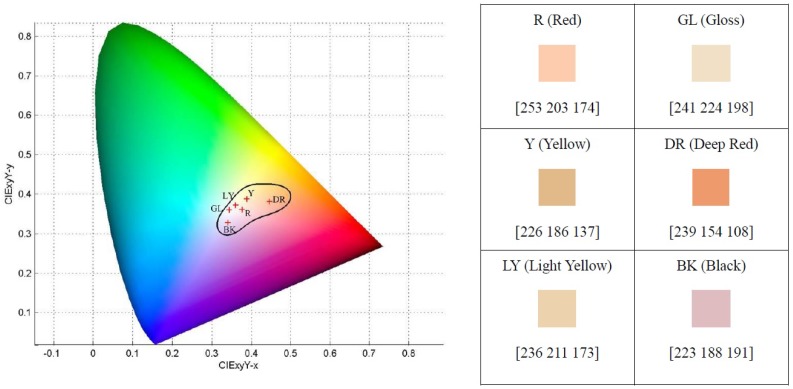
Facial color gamut.

**Figure 9:**
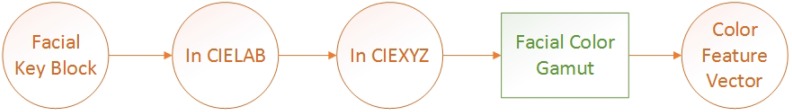
Facial key block color feature extraction procedure.

Figure 8 shows the facial color gamut in the CIEXYZ color space. In the CIEXYZ color space, the facial colors are bounded in a black region. In this figure, the right part depicts the six color centroids. Using the color gamut consisting of six color centroids, a color feature vector of length six is extracted from each facial key block. Figure 9 summarizes the color feature extraction procedure. More information about facial key block color feature extraction can be found in [12].

### 2.5. Classification

In NBDS, the ProCRC [25] is used as the classifier due to its effectiveness and speed. This subsection describes how ProCRC is applied to NBDS. Let $S=\left[S_{1},S_{2},\ldots ,S_{K}\right]\in R^{m\times n}$ denote the training samples, where $S_{k}$ is the training dataset from the $k_{th}$ class, where *m* and *n* represent the sample feature dimensionality and training sample number, respectively. A test sample $t\in R^{m}$ is classified using the ProCRC, which is calculated through Equations (5) and (6):

(5) $$\left(\hat{A}\right)=\arg\min_{A}{\{∥t-SA∥}_{2}^{2}+{\lambda ∥A∥}_{2}^{2}+\frac{\gamma }{K}\sum_{k=1}^{K}∥SA-S_{k}A_{k}{∥}_{2}^{2}\},$$

where *A* is the coefficient for which the training samples represent the test sample, and λ and γ are the scalars that balance the representation and coefficient:

(6) $$id\left(t\right)=\arg\min_{k}∥S\hat{A}-S_{k}{\hat{A}}_{k}{∥}_{2}^{2}.$$

Equation (6) labels the test sample *t* based on the solved coefficient $\hat{A}$ from Equation (5). The test sample belongs to the class whose residual value is the minimum. More details about the ProCRC can be found in [25].

## 3. Experimental Results

The experimental results are shown and discussed in this section. The setup of the experiments is first given followed by an evaluation of the system performance. In order to show the effectiveness of the sensor along with the ProCRC, four additional classifiers are compared. The four classifiers consist of two traditional classifiers (*k*-Nearest Neighbors (*k*-NN) [35] and Support Vector Machines (SVM) [36]) and two representation based classifiers (SRC and CRC).

### 3.1. Experimental Setting

To test our proposed system, a new dataset consisting of 119 BD and 595 H samples were collected from the Guangdong Provincial TCM Hospital, Guangdong, China in 2015. During image capture, any preparations of the facial skin regions were not required. We wanted to capture and analyze the raw images coming from the two key classes. The BD samples were diagnosed by medical professionals practicing Western Medicine using the traditional techniques mentioned in Section 1, where different forms of this disease were grouped into a single class (BD). There are two main kinds of BD in the dataset. Table 1 shows this information along with their corresponding sample numbers. The two sub-classes are Cerebral Infarction (CI) and other BD (OBD). OBD contains miscellaneous brain diseases, where the number of samples were not large enough to form its own specific class.

On the other hand, H samples were diagnosed through a blood test and other commonly used examinations. In order to decrease the affect caused by the unbalanced data, the H dataset is first randomly split into five equal parts (each part with 119 samples). Next, each H part and the whole BD formed a new dataset. Then, half (59+59=118) of the new dataset is applied to train NBDS and the remaining half (60+60=120) are used to test NBDS. Finally, the performance of NBDS is measured according to the mean of the five results.

It should be noted that all procedures performed involving human participants were in accordance with the ethical standards of the institution and/or national research committee and with the 1964 Helsinki declaration and its later amendments or comparable ethical standards.

In this paper, three measurements are applied to evaluate the system. The three measurements are Accuracy, Sensitivity, and Specificity [37]:

(7) $$\mathrm{Accuracy}=\frac{\mathrm{True}\;\mathrm{Classified}}{\mathrm{All}\;\mathrm{Data}\;\mathrm{Number}},$$

(8) $$\mathrm{Sensitivity}=\frac{\mathrm{True}\;\mathrm{Classified}\;\mathrm{Positive}}{\mathrm{All}\;\mathrm{Positive}\;\mathrm{Number}},$$

(9) $$\mathrm{Specificity}=\frac{\mathrm{True}\;\mathrm{Classified}\;\mathrm{Negative}}{\mathrm{All}\;\mathrm{Negative}\;\mathrm{Number}}.$$

According to Section 2.2, the facial image captured through the sensor is a size of W×H. In the system, W=768 and H=494. Based on Section 2.3, four facial key blocks are extracted from the four facial regions, where the size of each facial key block is set to be 64×64. The facial image captured by our specially designed sensor (refers to Section 2.2) is of size 768×576. Through some related experiments, 64×64 was chosen as the best size for the key blocks. Since a block with this size can represent its corresponding region very well. As LCB and RCB are symmetrical, there will only be slight differences between their color feature vectors. Therefore, in the following experiments, only three facial key blocks (FHB, LCB, and NBB) are employed. In order to further improve the performance of NBDS, all combinations of the three blocks are experimented.

### 3.2. NBDS Performance

The following experimental results were conducted on a PC with 8 i7-6700 CPU @3.40GHz processor, 16.0GB RAM, and a 64-bit OS. According to Section 2.3 and Section 3.1, all combinations of the three facial key blocks are applied in this system. The combinations consist of three single blocks (FHB, LCB, and NBB), three groups of two blocks (FHB+LCB, FHB+NBB, and LCB+NBB), and one group of three blocks (FHB+LCB+NBB). All seven combinations were experimented and the optimal group was selected. Hence, the system performance with various combinations were first measured. Based on Section 2.5, there are two parameters (λ and γ) in the classifier (ProCRC). Therefore, NBDS results applying different λ and γ values are next given, respectively.

#### 3.2.1. BD vs. H

In the following figures, the blue line represents accuracy, sensitivity is shown using an orange line, and the yellow line depicts specificity.

**Figure 10:**
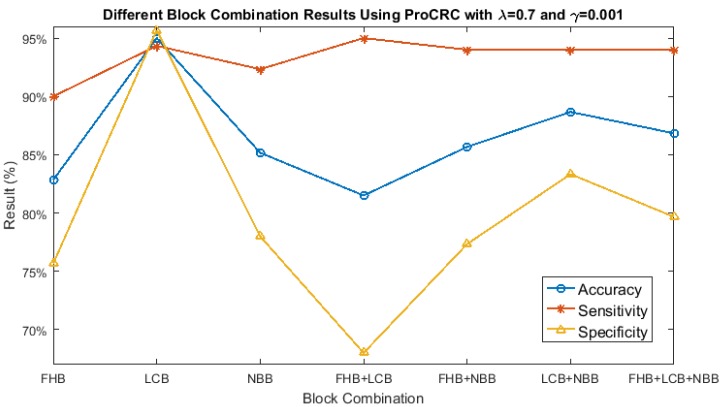
Different block combination results of Brain Disease (BD) vs. Healthy (H).

First, we show the different block combination results, the two parameters of ProCRC were first fixed, where λ equals 0.7 and γ=0.001. This is illustrated in Figure 10. According to this figure, all the accuracies were above 80%, where LCB obtained the highest accuracy of 95% with a sensitivity of 94.33% and a specificity of 95.67%.

**Figure 11:**
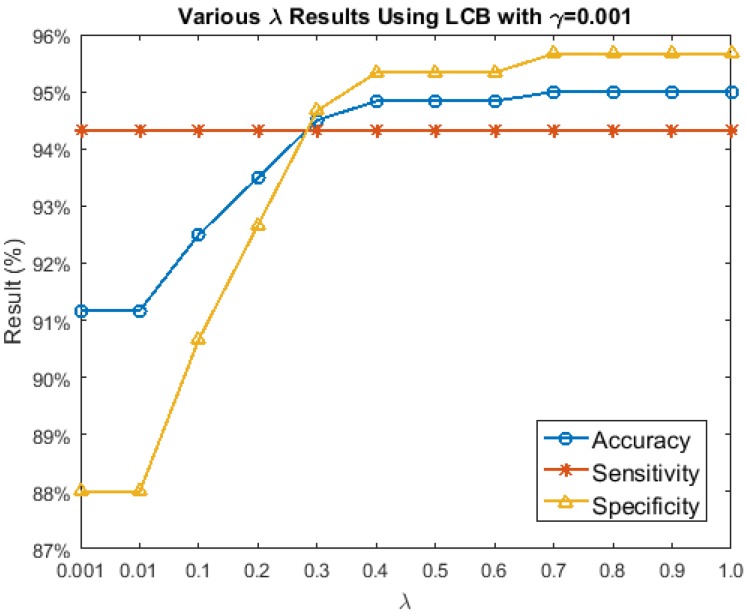
Various λ results of Brain Disease (BD) vs. Healthy (H).

Since LCB achieved the best result from the seven combinations, the following experiments will analyze different λ and γ values for this block. First, we fix γ=0.001 and let λ range from [0.001, 0.01, 0.1:0.1:1.0]. This result is shown in Figure 11. The sensitivities did not change with different λ values, while the specificities and accuracies increased with the increasing of λ. However, from λ=0.7 to 1.0, the specificities and accuracies remained the same. With LCB and γ=0.001, the best result was obtained with λ=0.7,0.8,0.9,1.0. Hence, in the following γ experiment, λ is fixed to be 0.7.

**Figure 12:**
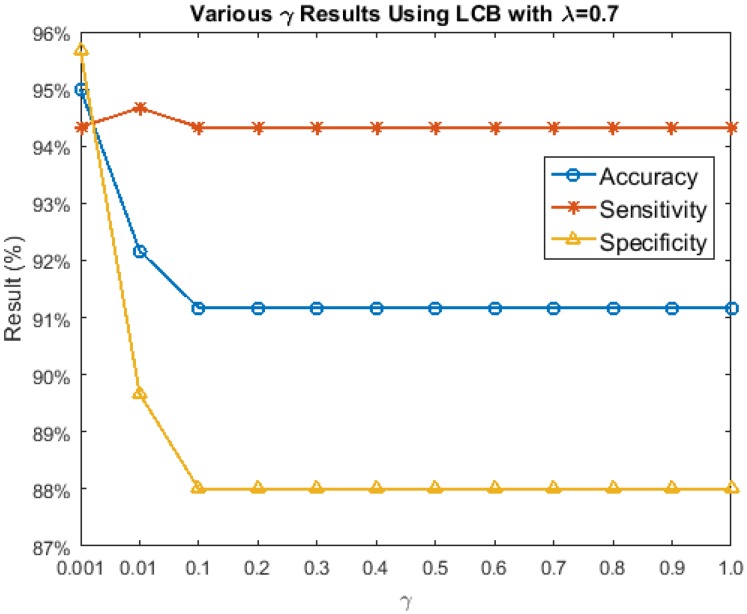
Various γ results for left cheek block (LCB).

Figure 12 depicts the results of λ=0.7 with γ ranging from [0.001, 0.01, 0.1:0.1:1.0]. Different from the λ results, the γ accuracies, sensitivities, and specificities did not change when γ≥0.1. From γ=0.001 to 0.1, the accuracies and specificities decreased, while the sensitivities remained somewhat stable, except when γ=0.01. Hence, the best performance of LCB is using ProCRC with a λ=0.7 and γ=0.001.

**Figure 13:**
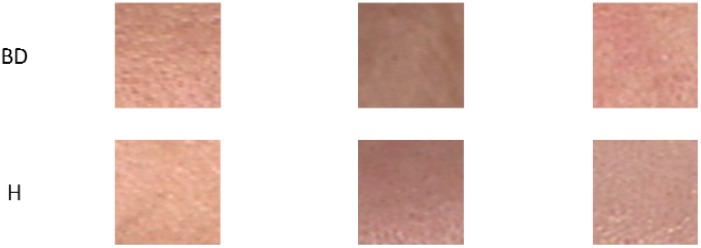
Three examples of LCB from BD and H, which cannot be distinguished with our naked eyes.

Figure 13 gives three typical examples of LCB (since it produced the best result) from BD and H. The top samples are from BD and the bottom blocks belong to H. Looking at the six blocks, we cannot recognize which one is from BD or H using our naked eyes. However, our proposed system (NBDS) can classify them correctly.

#### 3.2.2. Sub-Classes of BD vs. H

**Figure 14:**
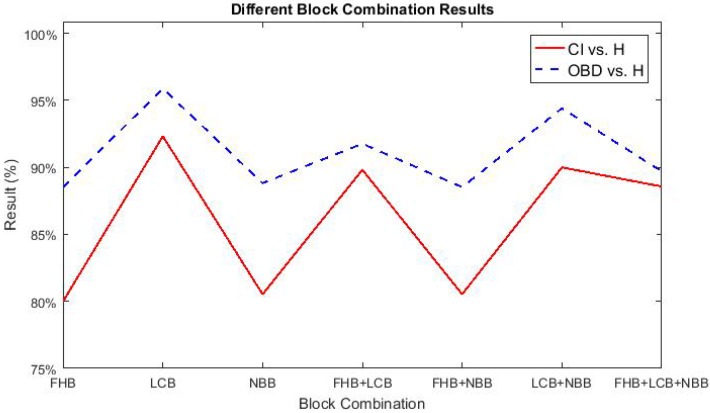
Different block combination results of BD sub-classes vs. H.

**Figure 15:**
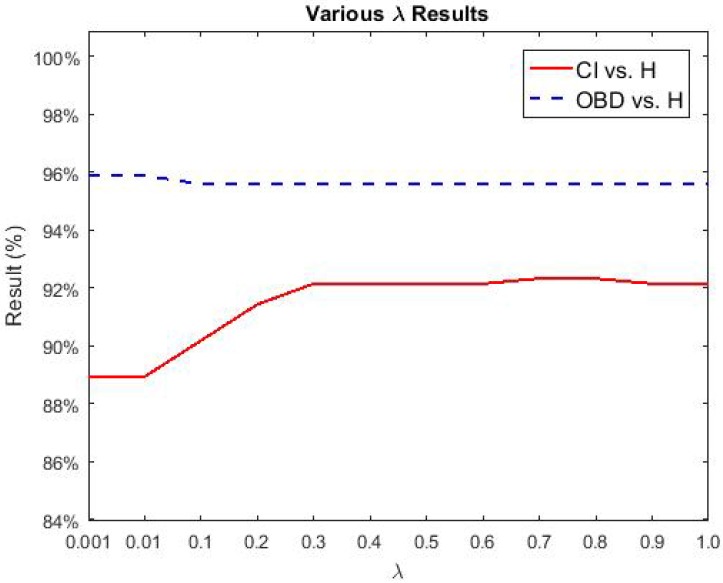
Various λ results of BD sub-classes vs. H.

**Figure 16:**
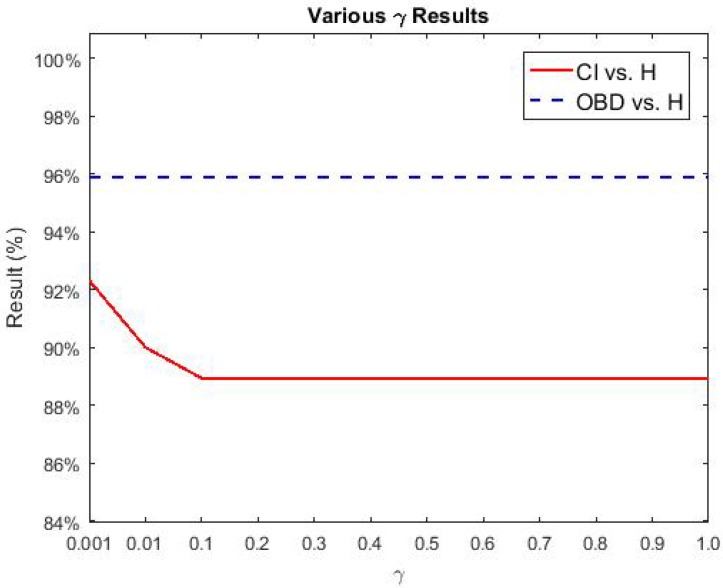
Various γ results of BD sub-classes vs. H.

To further test the performance of our proposed NBDS, additional experiments were carried out between healthy and the sub-classes of brain disease listed in Table 1. More specifically, they are CI vs. H and OBD vs. H. Applying the same experimental setup as described in Section 3.1, the following sub-section describes the output of NBDS. In the following figures (Figure 14, Figure 15 and Figure 16), the red solid line signifies CI vs. H and the results of OBD vs. H are represented using a blue dashed line. Both lines represent accuracy.

Figure 14 shows the different block combination results of CI vs. H and OBD vs. H. For CI vs. H, λ and γ were set to be 0.7 and 0.001, respectively, while both λ and γ equaled 0.001 for OBD vs. H. According to this figure, all of the accuracies are above 80%. Compared with CI vs. H, NBDS always obtained better accuracies for OBD vs. H.

The various λ accuracies of CI vs. H and OBD vs. H are depicted in Figure 15. According to Figure 14, LCB obtained the highest accuracies for CI vs. H and OBD vs. H. In this figure, for CI vs. H and OBD vs. H, LCB and γ=0.001 were fixed. Similar with Figure 14, the results of OBD vs. H were always the highest compared with the other classification task. For CI vs. H, the highest accuracy of 92.32% was reached, where λ=0.7. As for OBD vs. H, λ=0.001 obtained the greatest accuracy of 95.88%

With the above selected optimal block combinations and λ values, various γ results of CI vs. H and OBD vs. H are given in Figure 16. In this figure, the accuracies of OBD vs. H did not change, where once again the accuracies of OBD vs. H were higher than those of CI vs. H.

### 3.3. Classifier Comparison Results

The comparison results of the ProCRC and four other classifiers are given in this subsection. For all five of the classifiers, seven block combinations were applied. For the four classifiers to be compared with ProCRC, the parameters of each classifier were set according to experimentation that produced the highest accuracy. For *k*-NN, k=1, the linear kernel function is used in SVM; the balanced scalar λ=0.1 is set for SRC; and the CRC balance scalar λ equals 0.01.

**Figure 17:**
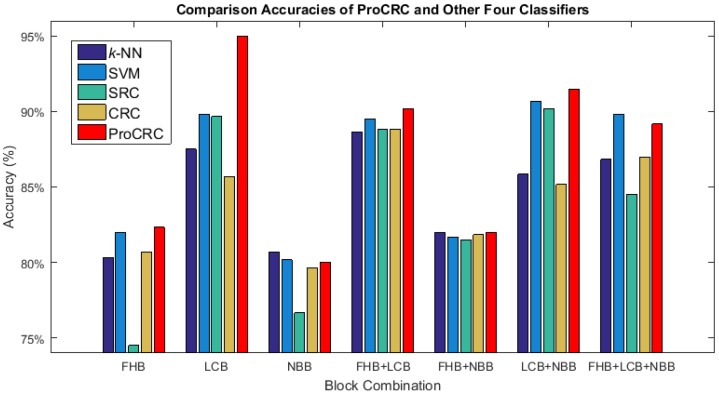
Comparison results of the ProCRC and the four classifiers.

The accuracies of the five classifiers are illustrated in Figure 17 with different block combinations. In this figure, the ProCRC performance is represented in a red bar. Except NBB, the ProCRC always achieved the highest accuracies among the five classifiers with different block combinations. Even when using NBB, the ProCRC accuracy was only 0.67% lower than the best results obtained by *k*-NN. The numerical results of the five classifier accuracies can be found in Table 2.

According to Section 3.2.1, the best results of NBDS was obtained by ProCRC with λ=0.7, γ=0.001, and LCB. In order to further show the best classification (ProCRC) performance, its experimental errors of five random rounds are shown in Table 3. In this table, the first row describes the round number and the second row represents its corresponding error. The final column gives the mean error of the five random rounds.

### 3.4. Running Time of NBDS

Another way to measure the proposed system is in its processing (execution) time. Table 4 shows the running time in seconds of the five classifiers with different block combinations. This tells us the average time it takes for a classifier to diagnose a block(s). As $l_{1}$-norm is time-consuming, the SRC with $l_{1}$-norm always took the longest time to perform classification, while the representation based classifiers using $l_{2}$-norm (CRC and ProCRC) were faster than the two traditional classifiers (*k*-NN and SVM). Compared with CRC, the ProCRC used less time to detect BD taking 0.6 milliseconds with LCB. In regards to the processing time of the other three components, image capture takes anywhere from 10 to 20 s (depending on the individual finding a comfortable position to rest their chin), facial key block extraction requires <5 s, while feature extraction can be accomplished in <10 s. This means the overall detection time of NBDS from input to output can be completed in less than one minute, which is a significant improvement compared to the traditional diagnostic methods.

## 4. Discussion

The NBDS was tested on a dataset consisting of only Chinese individuals. However, the underlying technologies in the form of the sensor, facial block feature extraction, and classification can be applied directly to detect brain disease in individuals from different nationalities. This is possible due to the fact that our sensor takes into account illumination variations and performs color correction. It allows an accurate facial image to be taken no matter their gender or race. Both the feature extraction (based on the results of facial block extraction) and classification components of NBDS are also invariant to the gender or the race of the individual being imaged.

Three facial key blocks were automatically extracted from various facial regions. Based on Section 3.2, in the seven combinations of the three blocks, NBDS using LCB (by itself) along with ProCRC obtained the best performance. In addition, NBDS can distinguish CI vs. H and OBD vs. H with accuracies of 92.32% and 95.88%, respectively (also using LCB and ProCRC), further proving the robustness and effectiveness of the proposed system. In terms of the classifier running times (refer to Table 4), ProCRC using LCB (0.6 milliseconds) did not achieve the fastest time of 0.4 milliseconds produced by NBB and LCB+NBB. However, when accuracy is factored in, NBB and LCB+NBB obtained 80% and 91.5%, respectively. Given the trade-off of an additional 0.2 milliseconds for an increase of 15% and 3.5% in accuracy, LCB was selected as the optimal block.

Compared with traditionally used brain disease diagnostic systems (such as those mentioned in Section 1), our NBDS has many advantages. First, the time it takes from examining an individual to reaching a diagnostic result is significantly improved. This allows more people to be tested on a daily basis and more frequently. Our device also emits lower and less harmful amounts of radiation. The way in which an individual is examined is non-invasive in nature, and is more akin to having one’s picture taken. Furthermore, the device’s size is relatively compact when compared to an MRI scanner, taking up less valuable space in health/medical facilities. That being said, an MRI, specifically the Cardiac Magnetic Resonance (CMR) relaxometry [38], has proven to be effective at detecting diseases before they occur. In addition, three-dimensional MRI images can be taken [39] to further analyze an object from all angles.

## 5. Conclusions

We all know that the brain is a very important organ and is the control center of the body. When it is damaged, it affects the daily life of the individual and can even cause death. The traditional diagnostic methods of brain diseases are not convenient or patient friendly. Therefore, we proposed a convenient and patient friendly system named NBDS to detect BD. NBDS consists of four components: (I) Facial image capture through the sensor; (II) Facial key blocks extraction; (III) Color feature extraction from each facial key block forming a feature vector; (IV) Classification of the feature vector using the ProCRC. In NBDS, different block combinations were experimented and LCB was selected as the most suitable block. Based on LCB using the ProCRC with λ=0.7 and γ=0.001, NBDS obtained an accuracy of 95% with a sensitivity of 94.33% and a specificity of 95.67%. Compared with traditional BD diagnostic methods, the average processing time of NBDS for a single individual is considerably shorter; in fact, the proposed system requires less than one minute. This allows more individuals to be examined than before, and also more frequently. Furthermore, since the proposed system is mostly automated (except for the first component), the process of BD detection can become less labor intensive, allowing medical personnel to focus on other tasks.

In the future, we will enlarge the dataset to include more samples. At the same time, more feature extractors and classifiers will be developed to detect BD. During data collection, other information regarding the individual, such as their physical condition and stress level, will be recorded to be analyzed at a later date in order to determine whether they play a role in distinguishing their health status via facial image analysis. In particular, tongue images, which can also be captured using our proposed device, will be analyzed and combined with facial color features to perform the detection of specific brain diseases.

## Figures and Tables

**Table 1:** Brain Disease (BD) types and its corresponding sample numbers.

BD Kind Name	Corresponding Sample Number
Cerebral Infarction (CI)	80
Other BD (OBD)	39

**Table 2:** Accuracies of the five classifiers.

	*k*-NN	SVM	SRC	CRC	ProCRC
FHB	80.33%	82.00%	74.50%	80.67%	82.33%
LCB	87.50%	89.83%	89.67%	85.67%	**95.00%**
NBB	80.67%	80.17%	76.67%	79.67%	80.00%
FHB+LCB	88.67%	89.50%	88.83%	88.83%	90.17%
FHB+NBB	82.00%	81.67%	81.50%	81.83%	82.00%
LCB+NBB	85.83%	90.67%	90.17%	85.17%	91.50%
FHB+LCB+NBB	86.83%	89.83%	84.50%	87.00%	89.17%

*k*-NN: *k*-Nearest Neighbors, SVM: Support Vector Machines, SRC: Sparse Representation based Classifier, CRC: Collaborative Representation based Classifier, ProCRC: Probabilistic CRC; FHB: forehead block, LCB: left cheek block, NBB: nose bridge block.

**Table 3:** Errors of the five random rounds using ProCRC.

Round No.	1	2	3	4	5	Mean
Error	0.0083	0.0250	0.1083	0.0583	0.0500	0.0500

**Table 4:** Running time (seconds) of the five classifiers.

	*k*-NN	SVM	SRC	CRC	ProCRC
FHB	0.1836	1.2646	4.4256	0.006	0.0026
LCB	0.0034	0.045	4.7146	0.0038	0.0006
NBB	0.0036	0.2904	4.768	0.0026	0.0004
FHB+LCB	0.067	0.3878	4.8236	0.003	0.0006
FHB+NBB	0.003	0.4258	4.5844	0.0024	0.0006
LCB+NBB	0.0032	0.3232	5.089	0.0026	0.0004
FHB+LCB+NBB	0.0032	0.6282	5.2658	0.003	0.0014
